# A Comprehensive Review of the Functional Potential and Sustainable Applications of *Aronia melanocarpa* in the Food Industry

**DOI:** 10.3390/plants13243557

**Published:** 2024-12-20

**Authors:** Jing Xu, Fusen Li, Meizhu Zheng, Li Sheng, Dongfang Shi, Kai Song

**Affiliations:** 1 Institute of Innovation Science and Technology, Changchun Normal University, Changchun 130032, China; xujingl@yeah.net (J.X.); lfs18844572435@163.com (F.L.); zhengmeizhu2008@126.com (M.Z.); 2 School of Life Science, Changchun Normal University, Changchun 130032, China; 3 Jilin Qiu Zhiyuan Ecological Technology Co., Ltd., Siping 136000, China; qzy_shengli@163.com

**Keywords:** *Aronia melanocarpa*, polyphenols, antioxidant activity, bioavailability, functional foods, sustainable development

## Abstract

*Aronia melanocarpa* (black chokeberry) is gaining attention in the food and health sectors due to its rich polyphenolic compounds and potent antioxidant properties. This paper provides a comprehensive review of the current research on the functional applications, bioavailability improvement strategies, and potential uses of *Aronia melanocarpa* in the food industry. The review highlights key developments in processing techniques, such as microencapsulation and nanotechnology, aimed at enhancing the stability and bioavailability of its active compounds. In addition, the paper explores the diversification of *Aronia* products, including juices, fermented beverages, and functional foods, and the growing market demand. The potential uses of *Aronia melanocarpa* leaves and by-products for sustainable production are also examined. Finally, the paper addresses the challenges of consumer acceptance, astringency removal, and the need for further research into the metabolic mechanisms and health benefits of *Aronia melanocarpa*. Future prospects for the *Aronia melanocarpa* industry, particularly its role in natural and sustainable food markets, are discussed, with an emphasis on innovative product development and the efficient use of by-products.

## 1. Introduction

*Aronia melanocarpa* (black chokeberry) is renowned for its rich polyphenol content and potent antioxidant capacity, which have gained it widespread application in the food and health sectors in recent years. Its fruits are abundant in antioxidants such as anthocyanins, proanthocyanidins, and phenolic acids, which effectively neutralize free radicals, thereby helping to prevent various diseases associated with oxidative stress [1,2,3,4]. These bioactive compounds not only endow *Aronia melanocarpa* with immense potential as a natural food colorant but also position it as a functional food ingredient with significant health benefits.

In the food industry, *Aronia melanocarpa* is widely used in the production of various products, including juices, jams, syrups, and teas [5,6,7,8]. Its deep purple fruits are particularly favored as natural colorants, especially in foods free of artificial additives. Additionally, *Aronia melanocarpa* has found extensive application in the dietary supplement sector, with its polyphenol extracts demonstrating significant effects in lowering blood glucose and lipids, as well as in anti-inflammatory and antihypertensive activities [9,10,11,12,13]. These applications not only cater to consumers’ growing demand for natural and healthy foods but also further enhance the plant’s prominence in the functional food market.

Current research on *Aronia melanocarpa* primarily focuses on its diverse applications as a functional food and the health benefits of its polyphenolic compounds. Its exceptional antioxidant properties make it an important source of natural functional ingredients, and related products are gradually expanding into areas such as dietary supplements and sports drinks [14,15,16]. Moreover, studies showed a close relationship between the polyphenolic compounds in *Aronia melanocarpa* and gut health, as they can modulate the gut microbiota, thereby improving overall health. Future research in this field will continue to explore these connections, particularly the link between gut microbiota and chronic disease prevention [17,18,19,20]. Furthermore, *Aronia melanocarpa* has garnered significant attention for its role in managing chronic diseases such as diabetes, cardiovascular diseases, and obesity. Its ability to lower blood sugar, cholesterol, and inflammation has been confirmed in several clinical trials [21,22,23,24,25].

As the anti-aging market expands, the antioxidant and free radical-scavenging properties of *Aronia melanocarpa* have gained increasing attention, particularly for their potential in beauty and anti-aging products. Simultaneously, researchers are working to optimize extraction techniques for its active compounds and improve the stability of these ingredients. This effort aims to maintain the efficacy of these functional components during processing and storage [26,27,28]. These trends will drive the broader application of *Aronia melanocarpa* in the food and health sectors, laying the groundwork for future discoveries of its potential health benefits.

Despite the great potential of *Aronia melanocarpa* in the food and health industries, there are still some issues and limitations in the current research, making further review and evaluation necessary. First, although numerous studies demonstrated the antioxidant, blood glucose-lowering, and anti-inflammatory properties of *Aronia melanocarpa*, most of these studies were confined to laboratory settings or animal models, with a lack of large-scale, long-term clinical trials in humans [29,30,31,32]. As a result, its health effects and safety in real-world settings have yet to be fully validated.

Additionally, the impact of different extraction and processing methods on the active compounds in *Aronia melanocarpa* requires further investigation. The stability of extracts during processing, storage, and in final products—especially the degradation and bioavailability of polyphenols—remains one of the key challenges for its practical applications [33,34,35,36]. Furthermore, while *Aronia melanocarpa* can be incorporated into a variety of functional foods, optimizing its nutritional profile and sensory attributes across different food matrices poses technical challenges.

Therefore, a comprehensive review of current research and applications is essential. In the following sections, we will systematically examine the research history of *Aronia melanocarpa*, its bioactive components, and its applications in the food sector. We will also analyze the evidence base for its health benefits and explore potential challenges and opportunities for future research and development to promote broader applications of this functional ingredient in the food industry.

## 2. Results

### 2.1. History and Global Spread of Aronia melanocarpa

*Aronia melanocarpa* (black chokeberry) originates from North America, where its medicinal properties were recognized by Indigenous peoples in ancient times. They utilized the berries and bark for medicinal purposes, such as creating astringents and other remedies. For example, the Forest Potawatomi Indians used *Aronia* berries to brew tea for treating colds, demonstrating the plant’s early medicinal potential [37]. This traditional knowledge laid the foundation for the broader dissemination and application of *Aronia melanocarpa*.

Over time, the distribution of *Aronia melanocarpa* expanded across the northeastern United States and the Great Lakes region, particularly in the higher elevations of the Appalachian Mountains. Residents of these areas mixed *Aronia* berries with fat, dried meat powder, and other fruits to produce a nutritious, long-lasting paste. This traditional food preparation method highlights the versatile uses of *Aronia* and opened new avenues for its application in food. By the early 20th century, large-scale cultivation of *Aronia melanocarpa* began in Siberia, Russia, and it gradually spread across Russia and Europe, where it gained popularity for its ornamental value and use as a natural colorant [38].

In European countries like Poland, Germany, Finland, and Sweden, the cultivation of *Aronia melanocarpa* quickly gained momentum, primarily due to its rich nutritional content and disease resistance [38]. In 1976, the former Soviet Union introduced *Aronia melanocarpa* to Japan, where the varieties “*Viking*”, “*Nero*”, and “*Aron*” became the most commonly grown types [39,40]. Subsequently, Sweden began cultivating *Aronia* in 1986, using it primarily as a source of natural food colorants. Other countries, such as Slovakia, followed suit and started cultivating *Aronia melanocarpa* as well [39,41].

China introduced *Aronia melanocarpa* in the 1990s, initially conducting experimental cultivation in the Liaoning province, which was followed by the introduction of several fruit-bearing and ornamental varieties. By 2015, the cultivation area had expanded to 12 provinces, covering a total area of approximately 600 hectares [42,43]. Thanks to *Aronia melanocarpa’s* adaptability, relatively simple cultivation requirements, and resistance to severe pest infestations—due in part to the fruit’s astringency—its global spread was rapid, and it is now widely cultivated across the world [42,43]. This global expansion demonstrates *Aronia melanocarpa’s* resilience in different climates and environments, as well as its increasing importance in the food industry.

### 2.2. Functional Properties of Aronia melanocarpa in Food

*Aronia melanocarpa* is rich in polyphenolic compounds, particularly anthocyanins, flavonoids, and phenolic acids, which confer it with notable antioxidant, anti-inflammatory, and antibacterial properties (Table 1). Although polyphenolic compounds generally exhibit low bioavailability during absorption and metabolism in the human body [44], *Aronia melanocarpa* demonstrates robust antioxidant activity through various mechanisms. These include scavenging free radicals, inhibiting reactive nitrogen species (RNS) and reactive oxygen species (ROS), restoring antioxidant enzyme activity, inhibiting pro-oxidants, and modulating cell signaling pathways [45,46]. These attributes make it an ideal functional food ingredient.

#### 2.2.1. Application as an Antioxidant

The polyphenolic compounds in *Aronia melanocarpa*, particularly anthocyanins, exhibit powerful antioxidant capabilities. Oxidative stress is closely linked to the development of numerous chronic diseases, including atherosclerosis, diabetes, and cancer. Incorporating *Aronia melanocarpa* extracts or its derivatives into food formulations can help enhance the antioxidant capacity of products, thus providing greater health benefits.

For example, *Aronia melanocarpa* has been widely used in functional beverages, yogurts, juices, and similar products. Its rich polyphenolic content not only boosts the antioxidant properties of these products but also imparts a unique color and flavor. Additionally, *Aronia melanocarpa* extract can serve as a natural antioxidant additive, extending the shelf life of foods by preventing quality degradation caused by oxidation [55,56].

#### 2.2.2. Application as a Natural Antimicrobial Agent

The potential of *Aronia melanocarpa* in antimicrobial applications makes it a promising natural solution for food preservation. Research by Svetlana M. Paunović and colleagues showed that *Aronia melanocarpa* extract inhibits a variety of common pathogens, including *Staphylococcus aureus*, *Klebsiella pneumoniae*, and *Escherichia coli*, and fungi such as *Candida albicans* and *Aspergillus niger* [57]. Inga Liepiņa et al. [58] also demonstrated that extracts of *Aronia melanocarpa* in different states (fresh, dried, and frozen) exhibit antimicrobial activity against Gram-positive bacteria, such as *Bacillus cereus* and *Staphylococcus aureus*, and inhibit the growth of Gram-negative bacteria such as *Pseudomonas aeruginosa* but show no inhibitory effect on fungi. Furthermore, it was confirmed that different processing conditions alter antimicrobial activity, with freezing reducing the antimicrobial effect. Drying or freezing the fruits leads to an increase in pH, although pH value does not serve as the determining factor affecting the antimicrobial activity of the extract. This antimicrobial activity can be leveraged to extend the shelf life of foods and reduce the use of preservatives, while also meeting consumer demand for natural, additive-free products.

*Aronia melanocarpa’s* antibacterial properties are particularly suited for preserving dairy, meat, and fruit and vegetable products. For example, adding *Aronia melanocarpa* extract to processed meat can significantly extend its shelf life and reduce spoilage. Magdalena et al. [59] applied *Aronia melanocarpa* leaf extract to typical spoilage and pathogenic bacterial strains found in meat and meat products and observed that it primarily affects bacterial growth parameters by reducing the growth rate and prolonging the lag phase. The results showed that *Lactobacillus monocytogenes* exhibited the strongest resistance, while the most sensitive bacteria were *Staphylococcus aureus* and *Clostridium perfringens*, with *Salmonella enterica* being the pathogenic bacterium of concern. Laura Tamkutė et al. [60] used supercritical CO2 extraction to defat *Aronia melanocarpa* fruit pomace and then employed ethanol and water for continuous extraction under pressurized conditions. The fruit pomace extract effectively inhibited foodborne pathogens and spoilage bacteria in actual pork and its by-products, significantly improving the safety and shelf life of meat products. Similarly, in fruit and vegetable beverages and fermented foods, *Aronia* extract can effectively control microbial growth, thereby maintaining the safety and flavor of the products [61].

#### 2.2.3. Application in Metabolic Health Functional Foods

Although *Aronia melanocarpa* has long been considered a lesser-known fruit, its health benefits are increasingly being recognized as the demand for healthy foods grows globally. *Aronia melanocarpa’s* unique ability to regulate metabolic disorders makes it an ideal ingredient for developing functional foods that promote metabolic health. Its polyphenolic compounds support the prevention and management of metabolic diseases, such as diabetes and hyperlipidemia, by improving insulin sensitivity, lowering blood glucose levels, and regulating lipid metabolism [62]. Additionally, the active components in *Aronia melanocarpa* inhibit key enzymes such as dipeptidyl peptidase-IV (DPP-IV) and α-glucosidase, thereby aiding in postprandial blood glucose control [12].

Given these metabolic health benefits, *Aronia melanocarpa* is now widely used in various products designed to promote metabolic health. For instance, *Aronia* juice and concentrated extracts are frequently included as key ingredients in functional beverages, allowing consumers to easily incorporate beneficial active compounds into their daily diets. Moreover, *Aronia melanocarpa* is commonly found in dietary supplements aimed at supporting diabetes management and preventing metabolic syndrome [63].

### 2.3. Strategies to Enhance the Bioavailability of Aronia melanocarpa

Although the polyphenolic compounds in *Aronia melanocarpa* offer significant health benefits, such as antioxidant, anti-inflammatory, and metabolic-regulating effects, their low bioavailability limits their widespread application in functional foods [64]. Bioavailability refers to the extent and rate at which active ingredients are absorbed and utilized by the human body. Polyphenols in *Aronia melanocarpa* are prone to degradation or transformation during digestion, which affects their stability and activity in the body. Therefore, enhancing their bioavailability has become a key focus in food science research.

#### 2.3.1. Microencapsulation Technology

Microencapsulation is one of the most effective methods for improving the bioavailability of polyphenols. This technique involves encapsulating polyphenolic compounds in a protective shell to prevent their premature degradation in the gastrointestinal tract. The protective shell is typically made of biocompatible materials such as proteins, lipids, or polysaccharides. Microencapsulation not only stabilizes the polyphenols in *Aronia melanocarpa* but also controls their release, ensuring a gradual release and absorption during digestion, thus enhancing their absorption in the small intestine [65].

For example, studies showed that *Aronia melanocarpa* polyphenol extracts processed with microencapsulation exhibit significantly improved stability in the digestive tract, resulting in a marked improvement in their bioavailability [66]. Furthermore, microencapsulation can reduce the bitterness and astringency of polyphenols, making them more palatable in functional food products, which in turn increases consumer acceptance [67].

#### 2.3.2. Nanocarrier Systems

In addition to microencapsulation, nanotechnology has shown great promise in recent years for improving the bioavailability of polyphenolic compounds. Nanocarrier systems, by loading polyphenols onto nanoscale carriers, significantly increase the surface area of the compounds, thus enhancing the absorption rate. Nanocarriers such as nanoemulsions, lipid nanoparticles, and polymer nanoparticles were used in the delivery systems of polyphenolic compounds [68].

Nanoemulsions, in particular, are a common carrier system that enhances the solubility and absorption efficiency of *Aronia melanocarpa* polyphenols. For instance, dispersing polyphenols into a stable oil–water nanoemulsion increases their permeability in the intestine and protects them from rapid degradation during digestion [69]. Additionally, nanoemulsion technology allows for the further optimization of bioactivity and absorption efficiency by adjusting particle size, carrier type, and surface modifications [70].

#### 2.3.3. Synergistic Effects with Other Bioactive Components

Combining *Aronia melanocarpa* polyphenols with other bioactive components such as lipids, proteins, or probiotics is another important strategy for enhancing bioavailability. Studies showed that polyphenols, when combined with lipid components such as medium-chain triglycerides, form complexes with higher absorption efficiency. Lipids help increase the solubility of polyphenols during digestion, thus promoting their absorption in the small intestine [71].

Similarly, combining polyphenols with proteins can form stable complexes, reducing their degradation in the gastrointestinal tract. For example, adding whey protein or soy protein to *Aronia melanocarpa* extracts has been shown to improve the stability and absorption efficiency of polyphenols in the body. This strategy not only enhances the bioavailability of polyphenols but also provides new ideas for the development of functional foods, especially in products that are high in both protein and antioxidants [72].

Moreover, probiotics are considered effective in enhancing the bioavailability of polyphenols. Certain probiotics can promote the metabolism of polyphenols in the gut, producing metabolites with higher bioactivity. This mechanism not only boosts the absorption of *Aronia melanocarpa* polyphenols but also enhances their regulatory effects on gut microbiota, further contributing to metabolic health benefits [73].

#### 2.3.4. Process Optimization and Extraction Technology Improvements

In addition to the above-mentioned techniques, process optimization and improvements in extraction technologies are crucial for enhancing the bioavailability of *Aronia melanocarpa* polyphenols. During extraction, factors such as solvent selection, temperature control, and time management all affect the extraction efficiency and activity of polyphenolic compounds. By selecting the appropriate extraction solvents (e.g., ethanol, water, or ethanol–water mixtures) and optimizing extraction temperature and time, it is possible to maximize the extraction of active compounds from *Aronia melanocarpa* while ensuring their stability during processing and storage [74]. Kaloudi et al. [3] performed a consecutive extraction of *Aronia melanocarpa* with acidified methanol three times and quantitatively recovered phenolic compounds. They found that 73% of the anthocyanins were located in the fruit skin, while 78% of the phenolic acids were found in the fruit flesh. The extract was then encapsulated in maltodextrin and Arabic gum using spray-drying, achieving an encapsulation efficiency of 88% for both total phenolics and anthocyanins. In recent years, ultrasonic-assisted extraction (UAE) technology has significantly improved the release efficiency of polyphenols due to its ability to promote cell wall disruption. Studies have shown that UAE can increase the yield of extracted polyphenols by 85% and significantly enhance the antioxidant activity of the extract [75]. Xu et al. [76] further optimized the UAE parameters (such as power, time, and temperature), resulting in a significant increase in the extraction efficiency of anthocyanins, demonstrating its potential for industrial-scale extraction. Furthermore, enzymatic and ultrasound-assisted extraction technologies have gained widespread application in recent years. Enzymatic extraction uses specific enzymes to break down the cell walls of *Aronia melanocarpa*, releasing more polyphenolic compounds while maintaining their structural integrity [77]. Dai et al. [78] studied ultrasound-assisted macroporous resin fixed-bed adsorption and desorption of anthocyanins and non-anthocyanin monomeric phenolics from *Aronia melanocarpa*, effectively removing organic acids and soluble sugars from crude extracts. The purified anthocyanins reached a purity of 48.8%, while the polyphenol purity was 78.7%. Additionally, microwave-assisted extraction (MAE) technology has gained attention due to its rapid heating and polarization effects, promoting efficient polyphenol extraction. Research shows that MAE at 300 W for 5 min, using a 50% ethanol solution, can yield a total polyphenol yield of 420.1 mg GAE/100 g [79]. Dong et al. [80] used 1-phenyl-3-methyl-5-pyrazolone (PMP) pre-column derivatization combined with high-performance liquid chromatography (HPLC) to analyze the monosaccharide composition of *Aronia melanocarpa*, providing a foundation for further study. Wang et al. [81] employed ultra-high-performance liquid chromatography-orbitrap tandem mass spectrometry (UPLC-Orbitrap-MS/MS) combined with GNPS molecular networking to rapidly analyze and accurately classify 42 components of *Aronia melanocarpa* fruits, providing a basis for the research and development of its functional substances. Moreover, Yang et al. [82] analyzed the polyphenol content data of 750 *Aronia melanocarpa* infrared spectra and then used mid-infrared spectroscopy combined with the CARS-RFR model for a rapid and accurate quantitative detection of polyphenols, offering theoretical and technical support for polyphenolic research. These technologies not only improve the extraction efficiency of polyphenols but also ensure their stability and bioactivity in functional foods [83]. Meanwhile, the application of nanofiber carrier technology is also emerging. By electrospinning, *Aronia melanocarpa* extract was loaded onto polyurethane nanofibers, and the resulting composite material exhibited excellent antioxidant properties, opening up new avenues for the high-value utilization of polyphenols [84].

#### 2.3.5. Research Trends

The number of published papers over time is a key indicator of the developmental trajectory of a specific research topic. It clearly reveals the evolution of research interest, making it essential for analyzing development trends and predicting future directions. Typically, a sustained increase in publications over time signifies that the field is continuously attracting extensive attention and in-depth investigation from scholars.

This investigation leveraged the WoSCC database, managed by Clarivate Analytics, to curate and scrutinize the relevant literature on *Aronia melanocarpa* up to 20 October 2024. The search strategy was refined using the keyword “*Aronia*” for publications between 1965 and 20 October 2024. To ensure the integrity and specificity of the analysis, the search was limited to English-language documents categorized as either “Article” or “Review Article”, resulting in a dataset of 976 documents. Further filtration was applied to distill the literature most pertinent to the research theme, followed by the download of comprehensive records and cited references.

Given the format constraints of the CiteSpace 6.1.R6 software, the literature was segmented into two subsets for processing: “download_1-500.txt” and “download_501-976.txt”. These subsets were subsequently prepared for bibliometric analysis using CiteSpace, which involved format conversion and parameter configuration for in-depth data examination and visualization. This approach enabled the visual representation of key research themes and trends within the *Aronia melanocarpa* field, thereby offering a comprehensive understanding of the field’s current state, developmental trajectory, and foundational theoretical underpinnings for future inquiries.

**Figure 1:**
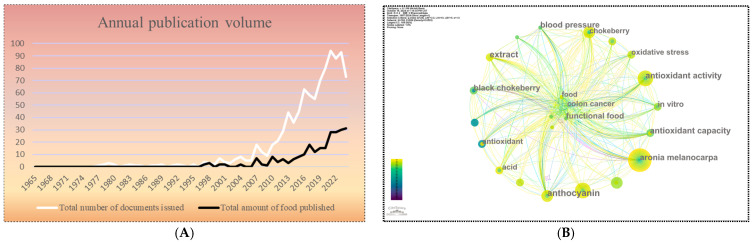
Annual publication volume of *Aronia melanocarpa* (**A**). Keyword clustering visualization analysis map based on Citespace (**B**).

As shown in Figure 1A, research on *Aronia melanocarpa* started relatively late, with the first article published in 1977. To date, a total of 976 articles have been published. In the early days, *Aronia melanocarpa* was primarily cultivated in home gardens as a source of berries and natural pigments. Due to technological limitations, the issue of its fresh fruits’ astringency and bitter almond flavor could not be effectively addressed, making the fresh fruit less acceptable to the general public [85]. Consequently, research before 2000 mainly focused on the transplantation and qualitative analysis of *Aronia melanocarpa’s* components.

A noticeable increase in publication volume occurred in the 2000s, with 76 papers published between 2000 and 2009, accounting for 7.787% of the total publications. Eleven of these focused on the quality analysis of *Aronia melanocarpa* fruits. During this period, researchers compared local *Aronia melanocarpa* varieties with imported ones through hybrid breeding and conducted a series of tests on the fruits, flowers, leaves, and bioactive substances to determine optimal fertilization, cultivation, and harvesting conditions. These studies identified varieties with the highest flavonoid and phenolic acid content, laying the foundation for further utilization of *Aronia melanocarpa’s* antioxidant capacity across various disciplines. Additionally, 69.736% of the 53 papers published from 2000 to 2009 analyzed the anthocyanins and antioxidant capacity of *Aronia melanocarpa*. As the taxonomic relationships of the *Aronia* species became clearer, the content and antioxidant performance of active components such as anthocyanins and phenolic acids became research hotspots, prompting more studies on their mechanisms of action in various pathological models.

Since 2010, the number of publications related to *Aronia melanocarpa* has steadily increased, peaking in 2021 with 94 publications. Of these, approximately 47 papers (50%) investigated the mechanisms of active substances such as anthocyanins and polyphenols in *Aronia melanocarpa* fruit extracts on cells and tissues. Another 9 papers (9.574%) explored the application and effects of the bioactive antioxidant capacities of by-products such as *Aronia melanocarpa* pomace. Additionally, 11 papers (11.702%) studied the impact of environmental and human factors on the biochemical effects of *Aronia melanocarpa* fruits.

Figure 1B illustrates a keyword analysis of 243 articles related to *Aronia melanocarpa* in the food field using Citespace. Keywords such as “*Aronia melanocarpa*”, “functional food”, “anthocyanin”, and “antioxidant activity” frequently appeared, indicating that, after 2010, with advances in science and technology, researchers moved beyond surface-level studies of the fruit and actively explored its beneficial compounds to contribute to human health. *Aronia melanocarpa* has been predominantly used as a processing ingredient, often combined with other fruits to produce juices [6], purees [86], jams, jellies, wines, syrups, teas, alcoholic or energy drinks, and yogurts [8,86]. It has also been widely used as an edible dye [87,88]. As the functional properties of *Aronia melanocarpa* continue to be developed and explored, its functional uses are gradually entering the public consciousness.

Despite the global expansion of *Aronia melanocarpa* applications and research, its industrial development in China still faces many challenges. First, the variety of seedlings is complex, and there is significant variation in fruit quality, leading to a wide disparity in the nutritional content, taste, and appearance of available varieties on the market. This presents challenges for consumer choice and market standardization [89]. Second, the level of deep processing of products is relatively low. Although *Aronia melanocarpa* has high nutritional value and health benefits, most of the products on the market remain in the primary processing stage, limiting product added value and market competitiveness. Furthermore, the issue of single-variety cultivation patterns also constrains the diversification and innovative development of the industry, making it difficult to meet the market’s demand for diverse products [89].

### 2.4. Processing Applications and Consumer Challenges of Aronia melanocarpa

#### 2.4.1. Market Demand and Astringency Removal Technology

With the increasing recognition of *Aronia melanocarpa’s* rich bioactive compounds and various health benefits, its market demand has continued to grow. In 2022, the global *Aronia melanocarpa* market was valued at approximately USD 720 million, and it is projected to expand to around USD 1.342 billion by 2030 [90]. Despite the astringent taste and bitter almond flavor of its fresh fruits, which limit consumer acceptance, various processing treatments and product development strategies have significantly broadened its application in the food industry.

*Aronia melanocarpa* is considered a valuable source of functional compounds with extensive health benefits [91]. However, the strong astringency caused by the high levels of soluble tannins, which bind to salivary proteins, has greatly limited its acceptance as a consumer product [91]. To improve the taste and nutritional value of *Aronia* juice, researchers have explored various methods for removing astringency. For instance, Li et al. [92] optimized a high-pressure, enzyme-assisted process to reduce tannin content in *Aronia melanocarpa* juice, achieving an 89.08% reduction in tannins while retaining 99.91% of anthocyanins. Zhang et al. [93] developed a unique fermented beverage by biologically fermenting *Aronia melanocarpa* juice with *Lactobacillus plantarum C8-1*, resulting in a nutritious and distinctively flavored product.

#### 2.4.2. Development of Juices and Blended Beverages

To further improve taste, researchers combined *Aronia melanocarpa* with other fruits. Hu et al. [94] developed a unique blended beverage by mixing *Aronia melanocarpa* with red raspberries, resulting in a flavor profile more acceptable to consumers. Jin et al. [95] created a well-received and nutrient-rich composite beverage by blending *Aronia melanocarpa* with hardy kiwifruit. Mao et al. [96] combined *Aronia melanocarpa* with blueberries to develop a drink with a balanced sweet-tart flavor and distinctive aroma. Additionally, Zhang et al. [97] produced a natural mixed drink with both nutritional and health-promoting benefits by using *Aronia melanocarpa* and purple carrots as raw materials. Liu et al. [98] developed a blended beverage of *Aronia melanocarpa* and black goji berries that offered a delicate, refreshing flavor, rich nutritional value, and antioxidant properties, meeting the demand for natural, healthy, and safe functional beverages.

In recent years, research on innovative beverages has further enriched the applications of *Aronia melanocarpa*. For example, Skąpska et al. developed a beverage made from *Aronia melanocarpa* with green tea and dandelion extract and treated it with high-pressure carbon dioxide (HPCD), which preserved the beverage’s high antioxidant content while enhancing flavor stability [99]. Wang et al. [100] combined *Aronia melanocarpa* with Chinese lantern plant husks to develop a novel functional drink with a unique fruity flavor and anti-inflammatory and anti-aging properties. Long et al. [101] developed an *Aronia melanocarpa* and peanut cake milk beverage with a rich milk fragrance and the distinct flavor of *Aronia melanocarpa*, offering a refreshing taste with a balanced sweet-sour profile, and the final product presented a milk powder color. Wang [102] produced a composite drink using “Southern Ginseng”—*Gynostemma pentaphyllum*— and *Aronia melanocarpa* as the main ingredients, optimizing the formula using response surface methodology to create a purple-red, clarified, sediment-free, mildly sweet and sour, and aromatic beverage. Xue et al. [103] blended *Aronia melanocarpa* with Sunshine Rose grapes and found that a 9:1 blending ratio with a 4-day maceration treatment resulted in the highest comprehensive score for the main components. The blend not only addressed the issue of insufficient sugar content in pure *Aronia melanocarpa* juice but also preserved the nutritional value of the fruit, while improving the sensory characteristics of the fruit wine. Sui et al. [104] brewed a dry fruit wine using *Aronia melanocarpa* and yacon, producing a gem-red wine that was clear, transparent, glossy, and balanced with a harmonious fruit and rich aroma, providing a full, smooth taste with an aftertaste. Lastly, innovative research also proposed a functional beverage combining *Aronia melanocarpa* juice with ginseng extract, which exhibited significant anti-inflammatory and antioxidant properties, showing potential benefits for metabolic health [31].

#### 2.4.3. Innovative Applications in Fruit Wines and Dried Fruits

As demand for healthy beverages grows, fruit wines—particularly those with antioxidant properties—are gaining popularity. Sun et al. [105] studied the effects of different pretreatment methods on the quality of *Aronia melanocarpa* fruit wine, finding that late-harvest fruit better retained its nutrients. This process was simple, cost-effective, and suitable for widespread adoption. Yao et al. [106] optimized fermentation conditions using single-factor and response surface experiments, successfully producing a high-quality fruit wine with a rich aroma, balanced sweetness and tartness, and enhanced antioxidant properties. Yang et al. [107] further optimized extraction techniques to create an *Aronia melanocarpa* wine rich in aromatic compounds. Zhang et al. [108] compared various fermentation methods and found that fruit wine fermented using cooked juice had superior alcohol content, fermentation performance, and antioxidant activity, with minimal loss of active ingredients during storage and the best sensory quality.

In the production of dried fruit, to meet the demand for healthier snacks, Li [109] used natural sweeteners to create low-sugar *Aronia melanocarpa* dried fruit, enhancing its quality and taste through electron beam irradiation pretreatment and optimized drying models. Xu [110] developed a flavorful dried fruit product with high bioactivity and antioxidant capacity by optimizing astringency removal and processing techniques, providing a scientific basis for dried fruit production.

#### 2.4.4. Integration of *Aronia melanocarpa* with Fermented Dairy Products

Yogurt, a fermented dairy product rich in nutrients, essential trace elements, and beneficial microorganisms, has long been regarded as a functional food with ideal health effects and enjoys widespread popularity. However, while dairy products, including yogurt, are known for their health benefits, they are not typically considered rich sources of antioxidants or key bioactive compounds such as polyphenols [111,112]. To enhance the health effects of yogurt, researchers have explored the potential of combining *Aronia melanocarpa*, a berry rich in polyphenols, with yogurt.

Zheleva, Nikolina, et al. [113] developed a natural functional food by combining bioactive phenolic compounds from wild cherry with buffalo yogurt, creating a product beneficial to human health. Boycheva, Svetlana, et al. [61] incorporated *Aronia melanocarpa* juice into sheep yogurt and found that the product not only had a faster coagulation rate and lower acidity than traditional natural yogurt but also contained higher levels of lactic acid bacteria and unsaturated fatty acids, enhancing its nutritional value and market competitiveness. These studies demonstrate that combining *Aronia melanocarpa* with fermented dairy products can significantly enhance product functionality and consumer acceptance, further expanding the application of *Aronia melanocarpa* in the food sector.

In recent years, researchers have begun to focus on the potential applications of *Aronia melanocarpa* in meat, poultry, as well as fish and shellfish products, particularly its potential as a functional additive. In meat products, polyphenolic extracts of *Aronia melanocarpa* have attracted attention due to their significant antioxidant and antimicrobial properties. For instance, applying *Aronia melanocarpa* extract to products such as pork sausages can not only significantly extend shelf life but also inhibit lipid oxidation and microbial growth, thereby improving the microbiological quality and sensory characteristics of the products [114]. Additionally, *Aronia melanocarpa* extract positively impacts the redness and water retention of meat products, making it a natural alternative to chemical additives. Meanwhile, Ren [115] demonstrated that adding 4% *Aronia melanocarpa* pomace to the feed of growing pigs is the optimal ratio. This not only improved the average daily weight gain of pigs and the proportion of beneficial bacteria in their intestinal contents but also significantly enhanced the anti-inflammatory and anti-apoptotic abilities of intestinal epithelial cells, highlighting its potential as an unconventional feed resource for development and utilization.

In poultry products, although relevant studies are relatively limited, preliminary results indicate that *Aronia melanocarpa* extract exhibits significant antioxidant and antimicrobial effects, effectively reducing the risk of oxidation and microbial growth during the processing and storage of poultry products such as chicken. These properties not only help improve food safety but also extend the shelf life and maintain the good sensory quality of poultry products. Furthermore, the addition of polyphenolic compounds may further enhance the nutritional value of poultry products, especially with regard to their potential in functional food production [116]. Bao [117] showed that adding *Aronia melanocarpa* pomace to the feed of egg-laying hens in the later stages of peak egg production had a significant beneficial effect on liver lipid metabolism and production performance, providing important support for improving poultry production performance.

In fish and shellfish products, the antioxidant capacity of *Aronia melanocarpa* is particularly suitable for reducing lipid oxidation, maintaining freshness, and extending shelf life. For example, by applying *Aronia melanocarpa* polyphenolic extracts to high-fat fish products such as salmon, studies showed a significant reduction in the formation of lipid oxidation products and an improvement in the sensory characteristics of the products [59]. Simultaneously, *Aronia melanocarpa* extract has demonstrated excellent antimicrobial and antioxidant properties in the storage of shellfish products like mussels and oysters, offering new possibilities for the high-value utilization of shellfish. Additionally, combining *Aronia melanocarpa* extract with other natural antioxidants or embedding it in food packaging materials to create dual-function antimicrobial and antioxidant packaging is an important direction for future development [114]. Furthermore, Wang et al. [118] found that adding 0.375% *Aronia melanocarpa* polyphenols to surimi products significantly improved water retention, gel properties, elasticity, and chewiness, forming a more stable surimi gel network structure. This further broadens the application of *Aronia melanocarpa* in the seafood processing industry.

These findings indicate that *Aronia melanocarpa*, as a functional additive, not only has a significant effect in traditional foods like fermented dairy products but also demonstrates wide application potential in meat, poultry, as well as fish and shellfish products. Future research can continue to optimize its extraction processes, evaluate its stability and efficacy in different food matrices, and explore its synergistic effects with other natural ingredients to further expand its applications in functional foods.

#### 2.4.5. Other Food Applications and Innovations

Bogdan Constantin Bratosin et al. [90] incorporated *Aronia melanocarpa* by-product powder into energy bars and found that it significantly enhanced the antioxidant activity and polyphenol content of the bars. Additionally, during 70 days of storage at room temperature, the bars exhibited inhibitory effects on fungi, yeast, and molds. This finding suggests that the development of *Aronia melanocarpa* by-products not only meets consumer demand for healthier foods but also promotes the advancement of functional food products.

In the confectionery industry, where synthetic dyes are associated with potential health risks, the use of natural colorants has become increasingly important. Aliona Ghendov-Mosanu et al. [119] explored the production of candies using *Aronia melanocarpa* extracts and powders. Their results showed significant improvements in the sensory characteristics, color saturation, and antioxidant activity of the candies. Wu et al. [120] developed effervescent tablets using freeze-dried *Aronia melanocarpa* powder and evaluated their antioxidant activity. The tablets demonstrated high levels of flavonoids and phenolic compounds, as well as favorable sensory properties, providing a solid foundation for the industrial production of effervescent tablets.

#### 2.4.6. Potential Uses of *Aronia melanocarpa* Leaves

In addition to the fruit, *Aronia melanocarpa* leaves have shown considerable potential for development. Studies indicate that *Aronia* leaves are rich in phytochemicals with antioxidant and anticancer properties. Dong-Wook Kim et al. [121] developed a high-bioactivity tea by using *Aronia melanocarpa* leaves as a substitute for traditional tea leaves. The study concluded that *Aronia* leaves should be regarded as a valuable raw material, rather than merely a by-product of fruit production, thus expanding the applications of *Aronia melanocarpa*.

#### 2.4.7. Prospects for Intelligent Food Packaging

As consumer needs evolve, traditional food packaging can no longer meet the demands of modern food safety and quality monitoring. Intelligent packaging, which can provide real-time data on temperature, humidity, and freshness, is gaining attention (Table 2). Such packaging can also detect food spoilage through color changes. Ayca Aydogdu Emir et al. [122] developed a colorimetric pH indicator film based on *Aronia melanocarpa*. The results indicated that the film could visually monitor food spoilage while exhibiting strong antioxidant activity to prevent food oxidation. This innovation highlights the broad potential of *Aronia melanocarpa* in food safety applications.

## 3. Future Development and Application Potential of *Aronia melanocarpa*

To fully unlock the potential of *Aronia melanocarpa* in the food industry, future research should focus on the mechanisms of action, safety, and efficacy of its active compounds. Ensuring the distinctive value of *Aronia melanocarpa* in both the food and pharmaceutical fields is crucial [91]. Technological innovation, particularly in the areas of modern biotechnology and nanotechnology, will play a pivotal role in enhancing the extraction efficiency of polyphenols while maintaining their bioactivity. These advancements can also improve the sensory characteristics of *Aronia melanocarpa*-based products, thereby increasing their market competitiveness. Currently, product development primarily focuses on the fruit, but the stems and leaves are also rich in nutrients, warranting further exploration [59]. Additionally, the comprehensive utilization of processing by-products, such as converting pomace into fertilizers or animal feed, will enhance resource efficiency, reduce waste, and promote sustainable development.

Moreover, the broad application of *Aronia melanocarpa* can be realized through the diversification of functional foods, including beverages, snacks, and dietary supplements, to meet the growing consumer demand for health-promoting products [120]. By delving deeper into the active compounds and their metabolic mechanisms, future products could be tailored to target specific health concerns such as cardiovascular health and diabetes prevention. As environmental awareness increases, efficient utilization of by-products not only boosts economic benefits but also drives the *Aronia melanocarpa* industry toward sustainable development, ensuring maximum resource utilization [4].

Looking ahead, *Aronia melanocarpa* is poised to strengthen its competitiveness in the global food market, particularly in response to the growing demand for “natural” and “sustainable” food products. By continuously optimizing extraction and processing technologies, the stability and efficacy of polyphenolic compounds in food processing can be ensured, further enhancing the value of *Aronia melanocarpa* as a functional food ingredient. Additionally, developing diverse products with specific health benefits, such as immune support, antioxidant activity, and gut health, will broaden the applications of *Aronia melanocarpa* and appeal to a wide range of consumers. Ultimately, the combined development of currently underutilized parts, such as stems and leaves, and the recycling of processing by-products will drive the *Aronia melanocarpa* industry toward more efficient, environmentally friendly, and economically sustainable growth [62].

## Figures and Tables

**Table 1:** Mechanism of active substances of *Aronia melanocarpa* in diseases.

No.	Disease/Condition	Active Compounds	Mechanism Studied	Key Findings	References
1	Infectious shock, neuropathic diseases, rheumatoid arthritis, and other autoimmune diseases	Cyanidin, Procyanidin B2, B5, and C1	Regulation of macrophage complement activity induced by lipopolysaccharides, inhibition of nitric oxide (NO) production, and the effect of these polyphenols on cell viability	*Aronia melanocarpa* procyanidins and procyanidin-rich fractions reduced NO production in lipopolysaccharide-activated RAW 264.7 macrophages	[47]
2	Autoimmune diseases and tumors	Polyphenols	Impact of pure or 1% pectin-enriched Aronia juice on markers of thymus degeneration in mature rats	Supplementation significantly stimulated CD3 + thymocyte subpopulations and delayed some age-related thymus changes at the microscopic level	[48]
3	Neuropathic diseases	2α, 3α, 23,24-tetrahydroxy-olean-12,19-diene-28-o-β-D-glucopyranoside, 2α, 3β, 23,24-tetrahydroxyoleanane-28-o-β-D-glucopyranoside, and 2α, 3β, 23,24-tetrahydroxyoleanane-12,19-diene-28-o-β-D-glucopyranoside	Inhibition of nitric oxide production by macrophages by isolated triterpene glycosides	Isolated compounds exhibited moderate inhibitory activity on nitric oxide production by macrophages	[49]
4	Neurodegenerative diseases	Anthocyanins	Evaluation of whether *Aronia melanocarpa* protects neuronal cells from glutamate-induced oxidative stress	High antioxidant activity of anthocyanins reduced glutamate-induced HT22 cell death	[50]
5	Inflammatory response	Quercetin and rutin from flavonol extracts	Study of antioxidant, immunomodulatory, and cytotoxic activities in lymphoblast RPMI-1788, with quercetin-rutin mixture reducing lipid peroxidation and enhancing phagocytic processes	Quercetin and rutin mixture positively influenced “respiratory burst” formation in neutrophils of healthy animals, leading to a reduction in reactive oxygen species activity	[51]
6	Hyperuricemia and gouty arthritis	*Aronia melanocarpa* fruit	Evaluation of *Aronia melanocarpa’s* ability to alleviate gouty arthritis and hyperuricemia in sodium urate crystal-induced acute gout rats and oxo-induced hyperuricemic mice	Reduced inflammatory cell counts in serum of acute gout rats, increased ankle joint space, inhibited interleukin (IL)-1β, IL-10, monocyte chemoattractant protein-1, and tumor necrosis factor-α levels; significantly lowered serum uric acid, blood urea nitrogen, and creatinine levels in hyperuricemic mice	[52]
7	Colorectal cancer	Anthocyanins	Investigation of the inhibitory effects of *Aronia melanocarpa* anthocyanins on Caco-2 cells and their suppression of malignant biological behaviors	Reduced cytoplasmic β-catenin and inhibited the expression of proteins in the Wnt/β-catenin signaling pathway	[21]
8	Muscle generation under chronic inflammation	Phenolic metabolites	*Aronia melanocarpa* treatment enhanced myogenesis in a model of chronic muscle inflammation	*Aronia melanocarpa* metabolites enhanced early myogenesis, characterized by increased expression of MymX and MyoG and formation of new myotubes; prevented muscle atrophy in a dexamethasone-induced model through muscle-specific ubiquitination prevention	[22]
9	Type 2 diabetes	*Aronia melanocarpa* extract	Regulation of glucose-lipid metabolism in type 2 diabetic rats through modulation of gut microbiota by *Aronia melanocarpa* extract	*Aronia melanocarpa* extract effectively modulated gut microbiota abundance, reduced colon tissue damage, increased body weight of diabetic rats, and lowered fasting blood glucose, LDL, and triglyceride levels	[53]
10	Alcoholic liver disease	Anthocyanins	*Aronia melanocarpa* anthocyanins alleviated ethanol-induced alcoholic liver disease by modulating the PI3K-Akt and Keap1/HO-1 pathways	Reversed α7nAChR and collagen I expression, downregulated PI3K-Akt and Keap1/HO-1 pathways	[54]

**Table 2:** *Aronia melanocarpa* intelligent food packaging indicating film.

Intelligent Indicator Film	Main Ingredients	Target	Functions, Effects, Advantages	References
*Aronia melanocarpa* Extract pH Colorimetric Edible Film	*Aronia melanocarpa* extract, starch biopolymer	Meat	The edible film turns pink under acidic pH and dark blue under alkaline pH. The color changes according to nitrogen released from spoiled meat. The film also exhibits strong inhibitory activity against Gram-positive bacteria, protecting food from degradation.	[123]
Chitosan-Based *Aronia melanocarpa* Extract pH Indicator Film	*Aronia melanocarpa* extract, chitosan	None	The film shows significant color differences within the pH 1–pH 10 range, with strong indication ability.	[124]
Anthocyanin-Based pH-Sensitive Packaging Film	*Aronia melanocarpa* anthocyanins, cassava starch, polyvinyl alcohol	Milk	Adding *Aronia melanocarpa* anthocyanins to the film significantly enhances its UV-blocking properties, making it more effective for monitoring milk freshness.	[125]
Intelligent Food Packaging Film with *Aronia melanocarpa* Extract	*Aronia melanocarpa* extract, chitosan, polyvinyl alcohol	Shrimp	The film shows excellent antioxidant, antibacterial, and pH-responsive properties. It can be used to monitor the storage of highly perishable shrimp and demonstrates great potential for the preparation of bioactive-enhanced intelligent food packaging films.	[126]
pH-Sensitive Dual-Layer Edible Film with *Aronia melanocarpa* Juice and Gellan Gum	*Aronia melanocarpa* juice, gellan gum, Ca^2+^ ions, pea protein, chitosan, Lactobacillus rhamnosus	Fresh pork	The edible dual-layer film, enriched with *Aronia melanocarpa*, functions as a freshness indicator. It offers an innovative approach to nondestructive freshness detection and has applications in 3D food printing with microencapsulation technology.	[127]
Multi-Material Composite Food Packaging Film with *Aronia melanocarpa*	*Aronia melanocarpa*, cellulose nanocrystals, grapefruit seed extract, polyvinyl alcohol, chitosan	None	The composite film exhibits significant color changes under different pH levels and strong antioxidant activity. It shows the highest antibacterial activity against E. coli (Gram-negative) and Listeria monocytogenes (Gram-positive), with excellent UV-blocking (95.5%), antioxidant activity (95%), pH sensitivity, and mechanical properties.	[128]
Anthocyanin-Based Intelligent Indicator Film with Three Types of Modified Starch	*Aronia melanocarpa* anthocyanins, cross-linked oxidized starch, acetylated distarch phosphate, hydroxypropyl distarch phosphate	Beef	The developed freeze-dried anthocyanin-based intelligent film demonstrated excellent stability and pH-responsive properties, with significant color changes during beef freshness monitoring.	[129]
Arrowhead Starch/Carrageenan/*Aronia melanocarpa* Anthocyanin pH-Sensitive Film	*Aronia melanocarpa* anthocyanin extract, arrowhead starch, carrageenan	Chicken wings	The film exhibits different color changes under varying pH and ammonia-sensitive conditions, reflecting the freshness of food.	[130]
Chitosan-*Aronia melanocarpa* Polyphenol Composite Coating Film	*Aronia melanocarpa* polyphenols, chitosan	Chilled pork	Inhibits microbial growth in food, extends shelf life, and delays pork spoilage.	[131]
Chitosan-*Aronia melanocarpa* Anthocyanin Composite Film	*Aronia melanocarpa* anthocyanins, chitosan	None	A green, eco-friendly intelligent food packaging material.	[132]
*Aronia melanocarpa* Anthocyanin Edible Film	*Aronia melanocarpa* anthocyanins, chitosan, zein, NADES	None	NADES, a novel natural plasticizer, shows excellent compatibility with anthocyanins and film components, superior to glycerol films. This significantly improves mechanical properties, moisture resistance, hydrophobicity, and antioxidant activity while reducing moisture content.	[133]
*Aronia melanocarpa* Pomace Polyphenol-Based Preservative Coating Film	*Aronia melanocarpa* pomace polyphenols, gelatin	Fresh pork	Pork samples without treatment spoiled by day 9, but *Aronia melanocarpa* pomace polyphenol-gelatin composite films extended the shelf life by approximately 3 days.	[134]

## Data Availability

The data that support the findings of this study are available from the corresponding author upon reasonable request.
